# Visual Feedback Dominates the Sense of Agency for Brain-Machine Actions

**DOI:** 10.1371/journal.pone.0130019

**Published:** 2015-06-12

**Authors:** Nathan Evans, Steven Gale, Aaron Schurger, Olaf Blanke

**Affiliations:** 1 Center for Neuroprosthetics, École Polytechnique Fédérale de Lausanne (EPFL), Lausanne, Switzerland; 2 Laboratory of Cognitive Neuroscience, Brain-Mind Institute, École Polytechnique Fédérale de Lausanne (EPFL), Lausanne, Switzerland; 3 Department of Neurology, University Hospital Geneva, Geneva, Switzerland; Duke University, UNITED STATES

## Abstract

Recent advances in neuroscience and engineering have led to the development of technologies that permit the control of external devices through real-time decoding of brain activity (brain-machine interfaces; BMI). Though the feeling of controlling bodily movements (sense of agency; SOA) has been well studied and a number of well-defined sensorimotor and cognitive mechanisms have been put forth, very little is known about the SOA for BMI-actions. Using an on-line BMI, and verifying that our subjects achieved a reasonable level of control, we sought to describe the SOA for BMI-mediated actions. Our results demonstrate that discrepancies between decoded neural activity and its resultant real-time sensory feedback are associated with a decrease in the SOA, similar to SOA mechanisms proposed for bodily actions. However, if the feedback discrepancy serves to correct a poorly controlled BMI-action, then the SOA can be high and can increase with increasing discrepancy, demonstrating the dominance of visual feedback on the SOA. Taken together, our results suggest that bodily and BMI-actions rely on common mechanisms of sensorimotor integration for agency judgments, but that visual feedback dominates the SOA in the absence of overt bodily movements or proprioceptive feedback, however erroneous the visual feedback may be.

## Introduction

Human action is associated with a sense of agency (SOA) characterized by the feeling that one’s movements and their consequences are self-generated and not externally produced [1–3]. Many behavioral [4,5], modeling [6,7], and neuroimaging studies [8–10] have investigated the brain mechanisms of the SOA. These studies have demonstrated that the SOA for bodily actions is attenuated when spatial and temporal conflicts are inserted between the action and its sensory consequences [11–15]. Theories based on predictive mechanisms link the SOA to internal forward models of motor control, where efferent-copy signals related to motor commands [16,17] are used to make predictions about the sensory consequences of the movement [18,19]. Discrepancies between the predicted and the actual sensory feedback weaken the evidence for a causal connection between action and feedback and reduce the SOA [20,21]. By contrast, cognitive or postdictive theories [22,23] hold that the SOA is based on the comparison between high-order movement intentions, anticipation, cognitive priors, and sensory outcomes.

Recent advances in brain-machine interfaces (BMI) have made it possible to decode cortical activity and generate machine-controlled actions without concomitant bodily action [24–26]. It is likely that BMIs will be increasingly exploited for purposes of neural rehabilitation (*e*.*g*. [27,28]), as well as by patients and healthy individuals who wish to restore and augment movement or communication capacity [29,30]. Yet, we currently lack empirical evidence and scientific understanding of the SOA for BMI-actions and knowledge of whether it relies on the same brain mechanisms as those described for body-driven actions.

**Fig 1:**
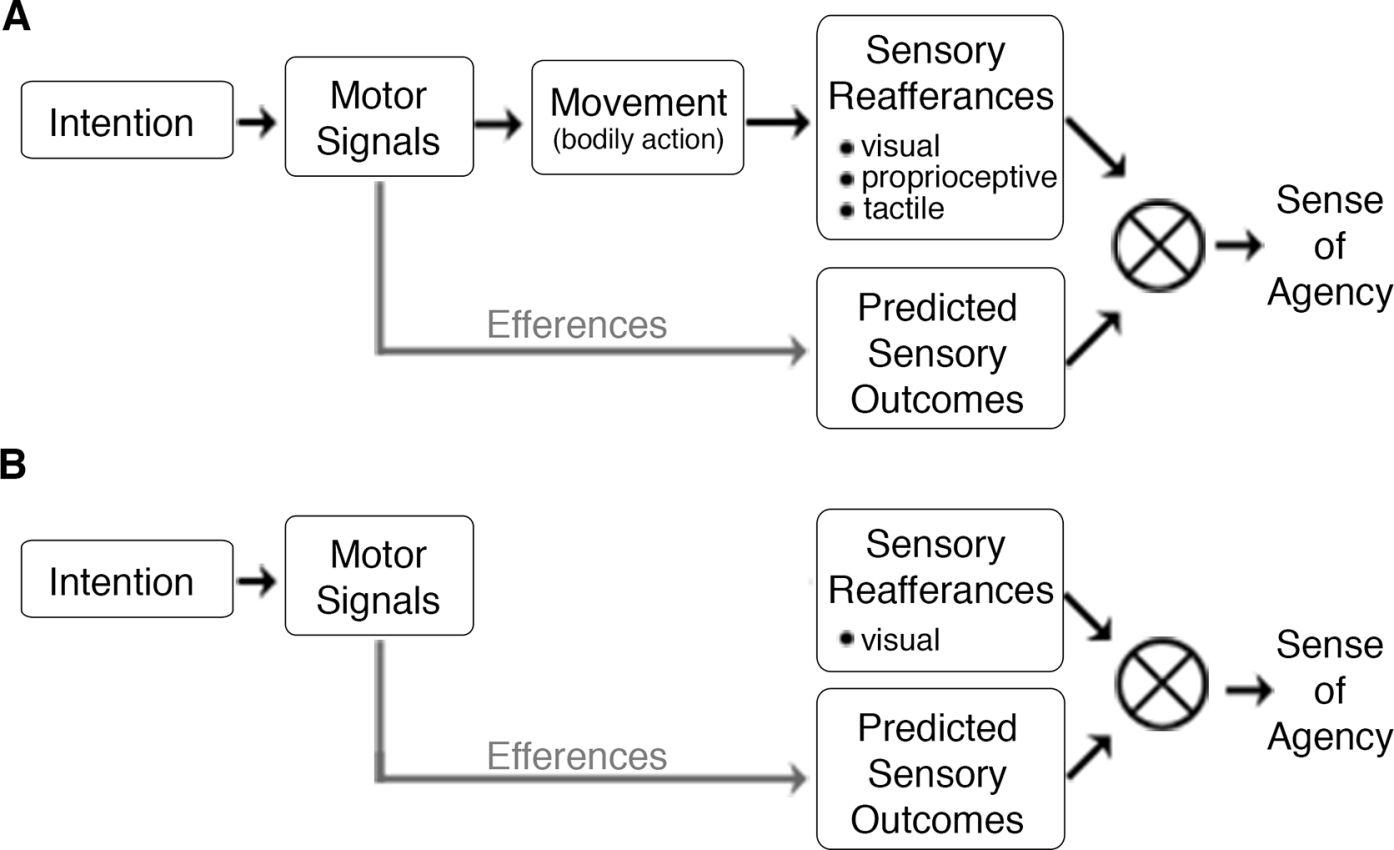
Schematic diagrams contrasting bodily and brain-machine actions in the context of predictive frameworks for the sense of agency (SOA). (A) Inspired by models of motor control, previous work on the SOA for bodily actions has proposed that a perturbed SOA stems from discrepancies between one’s predicted (efferent) and actual (re-afferent) sensory outcomes. These re-afferent signals may come from internal (*e*.*g*. proprioceptive and tactile signals stemming from muscle activity) or external sources (*e*.*g*. a visual flash on a screen). (B) In contrast to these previous paradigms, the current setup was designed to investigate the SOA for BMI-actions that lack bodily movement and the concomitant re-afferent tactile and proprioceptive signals. In this context, the SOA may depend on the comparison of one’s efferent motor commands (from motor imagery) or intended goal states with the re-afferent sensory outcomes. Schematic diagrams are based on [2].

We sought to examine the SOA associated with BMI-mediated actions, and to explore whether and how experimental manipulations used to study the SOA for bodily actions also impact BMI-actions. Using a motor-imagery-based, electroencephalographic (EEG) BMI [31], we introduced systematic conflicts between decoded motor signals and their resultant sensory consequences (visual feedback) and asked subjects to report their sense of agency. Thus, whereas prior studies introduced delays between *executed* movements and their visual consequences (motor-visual delay; *e*.*g*. [32]), the basic idea behind our study was to inject systematic delays between BMI-decoded cortical activity and its visual consequences (“neuro-visual delay”) and observe the effects on the SOA experience by the user. We also introduced spatial conflicts by manipulating the direction of cursor movement, which could be either congruent or incongruent with (opposite) the decoded movement direction. As participants in our experiments controlled the sensory consequences directly via brain activity, this paradigm allowed for investigation of the SOA in a novel setting where motor and movement-associated proprioceptive and tactile signals were absent (Fig 1).

## Materials and Methods

Two experiments were conducted with independent subject samples. For study 1, we tested the SOA for BMI-actions by introducing a range of neuro-visual delays. In study 2 we aimed to reconfirm findings in a different study sample and extend our investigation of the effects observed in study 1. Thus, for study 2 we utilized shorter neuro-visual delays, and also carried out a control experiment to verify that BMI control was performed without overt muscle contractions.

### Subjects

A total of 15 subjects were tested: Eight healthy, right-handed participants were recruited for study 1 (six males; aged 26.5 ± 3.5 years, *mean ± SD*), and an independent sample of seven healthy participants (seven males; 1 left-handed; aged 26 ± 2.3 years) was recruited for study 2.

### Ethics Statement

All participants provided written informed consent prior to participation and the present studies were undertaken in accordance with the Declaration of Helsinki and approved by the local ethics research committee at the University of Lausanne.

### Electroencephalography (EEG)

A 27-channel electroencephalography (EEG) montage was sampled at 128 Hz (*g*.*tec*, Schiedelberg, Austria) and data were saved and processed in real-time using a custom Simulink model (*Mathworks*, Natick, Massachusetts, USA). The electrode grid was centered over sensorimotor cortex, grounded with an additional electrode placed on the forehead, and re-referenced to an electrode attached to the right earlobe. Electrode placement and real-time data processing methods are described in detail in [31].

### Electromyography (EMG)

For study 1, we monitored participants while they were performing the task, to make sure that they were not making any overt movements, and regularly reminded them to remain motionless. However, in order to more objectively verify that muscle contractions were not involved in cursor control, we measured EMG activity in study 2. For study 2 we also added one additional experimental block of 40 trials (20 left hand; 20 right), where participants were asked to physically clasp their left or right hand according to the directional cue. EMG activity was sampled at 128Hz from electrode pairs placed on the left and right forearm flexor muscles midway between the wrist and elbow. The EMG signals were rectified and an average amplitude ratio (motor imagery divided by the maximum voluntary contraction; MVC) was constructed separately for left- and right-cued trials. Further details on data processing and analysis can be found in [33].

### Brain-machine interface training procedure

#### Phase 1: Training without visual feedback

Participants first trained to perform a lateralized motor imagery task without visual feedback [34]. Participants sat at a table with their hands on their laps (palms up), occluded by the table. They were instructed to relax and to avoid blinking and other body movements. A black fixation cross was first presented for 2000ms. Next, as a preparation cue, the fixation cross turned red for 500ms and was then overlaid with a left or right arrow for 1250ms indicating whether participants should imagine clasping their left or right hand, respectively. Following the directional cue, a fixation cross was presented for 4.25s while participants performed motor imagery. An experimenter visually assured compliance with the instructions throughout the experiment and provided reminders throughout the experiment in case of negligence.

#### Phase 2: Training with visual feedback

Coefficients were computed for a binary linear classifier discriminating between left and right imagined hand movements (see *EEG data processing*, below) and used in a second training phase with visual feedback. This training phase was used for the participants to learn the temporal and spatial dynamics of the relationship between their imagined movements and the movement of the visual cursor. The presentation of the stimulus was the same as for the task without visual feedback, except that following the directional cue, the fixation cross and cue disappeared and were replaced by a horizontally-oriented, rectangular cursor that participants were instructed to move to the cued edge of the screen using the trained motor imagery association. The cursor was controlled by real-time classifier output until the cursor touched one of the edges of the screen, or until 6s had passed without reaching either edge.

In order to better estimate chance performance, using the set of possible cursor velocities, we computed the minimum and maximum theoretical time to reach an edge (1.48s and 66.41s, respectively). We performed a simulation of 10,000 iterations where the cursor moved according to a random-walk and found it to never reach a target (mean absolute cursor distance traveled as a percentage of the target: 0.071 ± 0.054%; maximum distance traveled in an iteration as a percentage of the target: 32.29%) within the time allotted. By contrast, our empirical data showed that participants were able to control the cursor to reach a target in 3.31s ± 0.48 for study 1 and 3.25s ± 0.40 for study 2 (average across trials, experimental conditions and subjects).

After each training block of 40 trials (20 left cues, 20 right), proficiency in controlling the cursor was estimated by computing the real-time classification performance (percentage of time steps classified in the direction of the cue) on a trial-by-trial basis. If participants were unable to achieve >75% mean performance across trials in the training block, or did not verbally report that they felt able to move the cursor in the desired direction, the full training procedure was repeated until these criteria were met (generally 1 to 4 training blocks of 40 trials; study 1: 80 ± 37 trials *mean* ± *SD*; study 2: 80 ± 56 trials). This procedure was repeated up to five training blocks, after which participants still unable to sufficiently control the cursor were dismissed before participating in the main experiment. We elected to dismiss participants if they were unable to control the BMI during the training phase in order to more closely follow studies investigating the SOA for body movements, where participants are generally able to perform the required motor task (*e*.*g*. [12,14,35]).

### Experimental protocol and measurements

**Fig 2:**
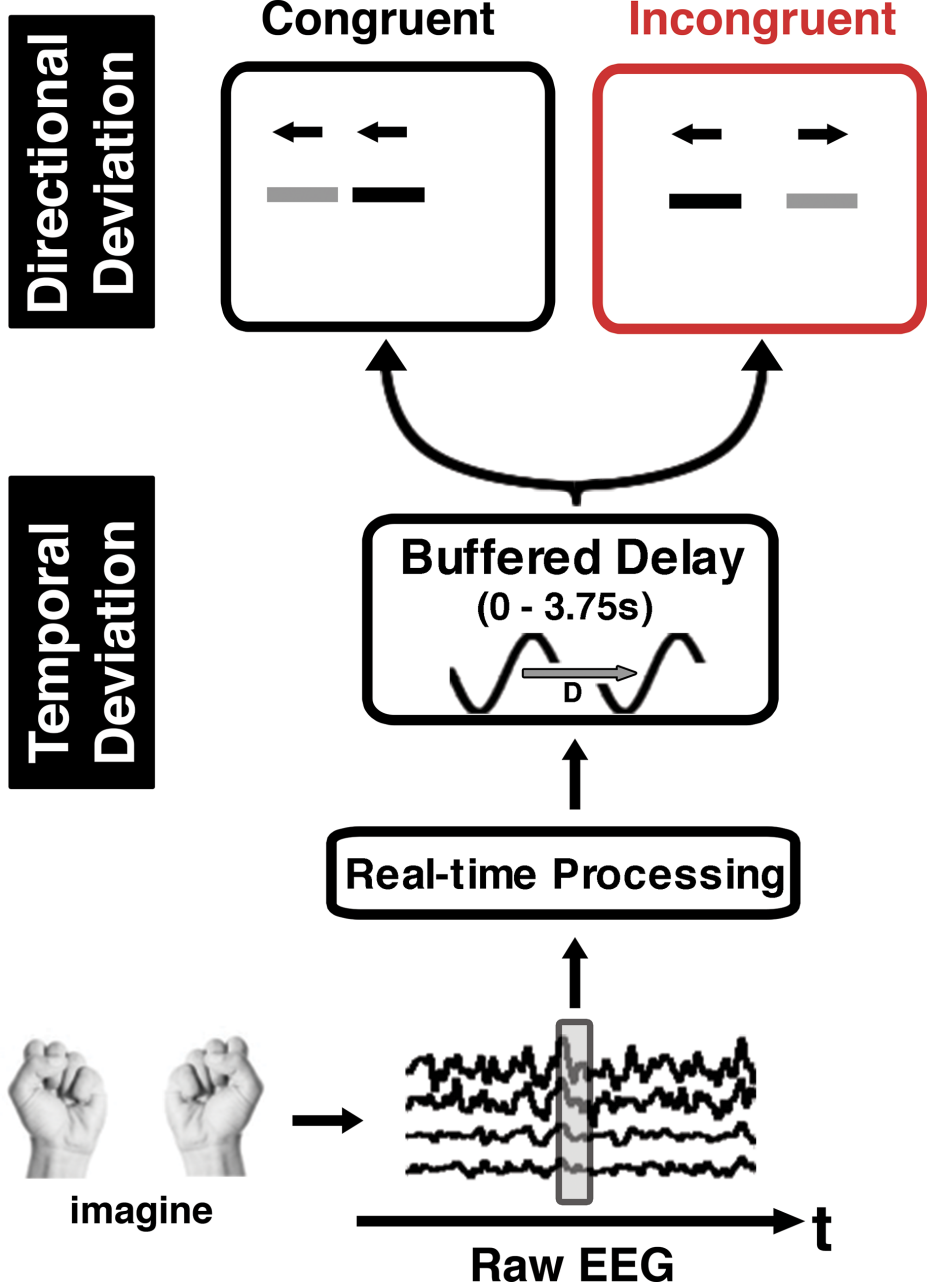
Task and experimental manipulations. Participants were asked to imagine clasping their left or right hand. EEG signals were measured, processed, and decoded in real-time in order to displace a cursor on a monitor to the left or right. For each trial of cursor control, one of six temporal deviations was applied using a buffered delay on the decoded motor signal. Additionally, a directional deviation was applied to the cursor position such that in half of the trials, the coupling between the classifier output and the cursor displacement was reversed (incongruent; red) from the learned association (congruent; black). For instance, for incongruent trials, left imagery resulted in right cursor displacement.

The presentation of the stimulus in the main experiments followed that of the training blocks with visual feedback (see *Training with visual feedback*). In both studies the visual consequences were experimentally manipulated by inserting a buffered delay into the real-time classifier output (Fig 2, *bottom*). Six delay conditions were tested in study 1 (0 to 3750 ms at 750 ms intervals). As the SOA is generally tested for shorter motor-visual delays, we tested a range of sub-second delays in study 2 (0 to 1000ms at 250ms intervals and 3750ms). Thus, in both studies, the cursor position was updated at every time step and was displaced according to classifier output associated with the brain activity from the current time step, or up to 3750 ms prior to the current time step. In study 1, an additional manipulation of cursor direction was made: for incongruent trials, classifier output was multiplied by -1, resulting in an inverted velocity mapping between classifier output and cursor position with respect to the learned directional association (Fig 2, *top*). Thus, for example on incongruent trials where the participants imagined left hand clasping, the cursor moved to the right, whereas for congruent trials the cursor moved to the left (the direction of the imagined movement).

An additional control condition (henceforth referred to as the ‘random-feedback’ condition) was included in both studies in which the cursor moved on a random trajectory decoupled from the neural activity. We used the following algorithm to generate the random trajectory: at a random interval (uniformly sampled between 250 and 1500 ms), the cursor velocity was randomly sampled (with replacement) from the same set of velocities used for cursor feedback in the other experimental conditions. As the ‘random-feedback’ condition was the same for both study 1 and study 2, all data analyses for this condition were performed on data collapsed across all participants from both studies (resulting in N = 15; 288 trials).

Trials were presented in experimental blocks (study 1: 5 blocks of 13 conditions x 3 trial repetitions; study 2: 4 blocks of 6 conditions x 7 repetitions + 1 block of 40 trials for electromyography). Within each block of trials, experimental conditions were randomized and interleaved. Participants were instructed to imagine clasping the cued hand, even if the cursor was not moving as desired.

#### Sense of agency measurement

SOA was gauged on a trial-by-trial basis with a yes-no response to the statement: “*I felt as if I was controlling the cursor I saw on the screen*.” Responses were transformed into a percentage of yes answers, resulting in a single percentage per subject, per experimental condition.

#### BMI classification performance measurement

Classification performance was taken as the percentage of time samples that the classifier output corresponded to the cued direction, regardless of where the (potentially) manipulated visual cursor was displaced. Thus, classification performance is also an indicator of the amount of time that participants spent in the brain state associated with the learned coupling between BMI-action and consequence.

### Statistical and psychometric analyses

Differences in SOA responses and classification performance across experimental conditions in study 1 were assessed using a 2 x 6 repeated-measures ANOVA with factors Congruence x Delay. After observing a significant interaction, we tested for the main effect of delay separately for congruent and incongruent trials with 1 x 6 repeated-measures ANOVAs. For any significant main effects we used post-hoc two-tailed, paired t-tests to assess differences across individual experimental conditions.

#### Trial-by-trial psychometric analyses

In order to test for an explicit relationship between SOA and the percentage of time that the cursor was displaced toward the cued direction, trials were pooled across participants and binned (4% width) such that each trial’s SOA response was paired with the percentage of time the cursor had moved in the cued direction. We elected to bin the data across participants to increase the robustness of the psychometric fits, after observing that individual subject SOA distributions for the main experimental conditions were similar to one another. Data were pooled separately in four conditions: 1) congruent, no delay; 2) congruent, high delay (3.75s); 3) incongruent, no delay and 4) incongruent, high delay. For study 2, only the first two conditions were used. Psychometric curves were then fit to the binned group data in these four conditions using binomial, logistic regression (glmfit logit function, Matlab, Mathworks, Natick, Massachusetts, USA; *e*.*g*. [32]).

For each of the four conditions, two measurements were collected from the best psychometric fit. First, the point of subjective equality (PSE) was taken as the classification percentage closest to 50% SOA. Second, we measured the sensitivity (slope) of the psychometric fit [36]. Standard errors on these values were obtained using the bootstrap method with 10,000 iterations and statistical differences across conditions were assessed using a one-tailed bootstrap test [37].

### EEG data processing

To classify the real-time EEG signals, offline classifiers were built for each subject from training session data to distinguish between left and right motor imagery patterns. Real-time EEG signals for each electrode were individually weighted using common spatial patterns [38] to create a feature vector and were finally subjected to linear discriminant analysis in order to generate the left or right velocity of the visual cursor. The following subsections describe this data processing in further detail.

#### Offline classifier computation

EEG data from the motor imagery period of the classifier training session were visually inspected for eye blink and muscle artifacts and trials with artifacts were removed. To further increase the robustness of the classifier in study 2, artifact-free trials were combined from two classifier-training sessions (in contrast to one training session for study 1). Thus, the number of artifact-free trials used to train the per-subject classifiers was 38 ± 2 (*mean* ± *SD*) for study 1 and 51 ± 14 for study 2. Of the remaining artifact-free trials used to train each participant’s classifier, approximately half (53.4% ± 1 SD across participants) were left and half were right. Note that due to this slight imbalance of left and right trials, all statistical tests were adjusted to account for a classification performance chance level of 53.4% ± 1 SD.

Data from the training session were used to compute common spatial patterns (CSPs) for each subject [38]. In study 1, CSPs were computed for motor imagery data from a fixed window (0.25–1.25s following the directional cue). In study 2, we further optimized the CSPs by selecting, for each subject, the window position that maximized the classification performance on the training set (from a 1.5s window at 0.5s intervals, between 0.25–4.25s following the cue).

Next, feature vectors were constructed by bandpass filtering the raw EEG in the mu (sensorimotor alpha oscillations; [34]) and beta frequency bands (8–30 Hz), reducing the dimensionality of the data by re-projecting it through the first two and last two CSPs, and then computing the log variance across a sliding window of timeframes (*see below*) in each of these four dimensions of the filtered signals. The per-subject classifiers (linear discriminant analysis; [39]) and per-subject CSPs were only computed during the classifier training phase without visual feedback and were held fixed throughout the remainder of the experiment.

#### Real-time preprocessing, classification, and visual feedback

Real-time EEG data were bandpass filtered, projected through the offline-computed CSPs, and log variance was taken using the Simulink model. Visual feedback was provided in the form of a rectangular cursor that could move to the left or right of its current position with a velocity proportional to the magnitude of the distance of the feature vector from the linear decision boundary [33,40]. The cursor trajectory was additionally smoothed by taking a sliding average (1s window) of the log variance. Thus, features (average log variance across this time window for each of the four CSP dimensions) were computed for each EEG sample and fed to the classifier, resulting in classifier output that translated to a new cursor position every ~8ms.

#### Additional verification of BMI control signals

**Fig 3:**
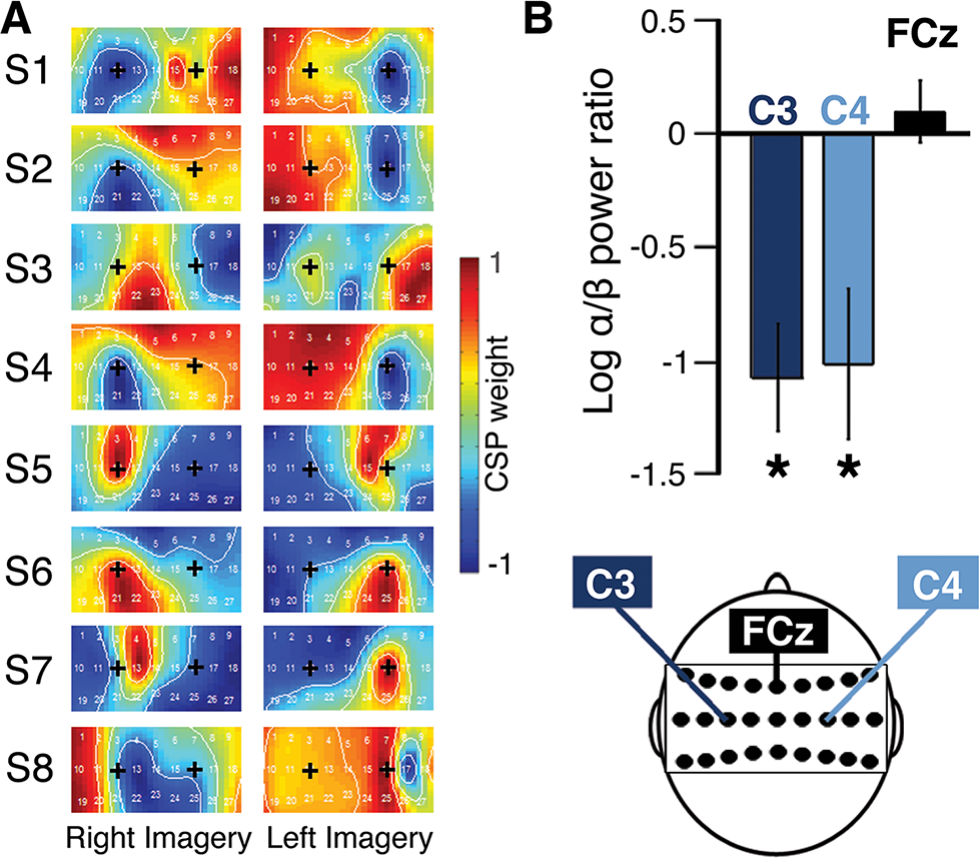
Cursor control driven by bilateral sensorimotor mu- and beta-band modulations. (A) Normalized, per-channel common spatial pattern weights for each electrode during left and right motor imagery for each participant (S1-S8) collected during the BMI training period (without visual feedback). On each topographic map, ‘+’ labels are placed over electrodes C3 and C4 for orientation. (B) Contralateral divided by ipsilateral log power ratios for 8–30 Hz oscillations at electrodes C3, C4, and control electrode FCz. * indicates significant suppression (*P < 0*.*05*; *two-tailed T-test*) with respect to a log power ratio of 0 (*i*.*e*. no difference between ipsilateral and contralateral power). Error bars represent SE of the mean.

To ensure that participants used sensorimotor rhythm modulation rather than non-EEG artifacts to control the cursor, we computed topographies of the statistically derived common spatial pattern filter weights for single subjects (Fig 3A; for methods on the computation of the weights see *Offline classifier computation*, above). The weights were normalized between -1 and 1 and projected for each channel onto a topographical EEG map (*e*.*g*. [31, 38]).

We verified the presence of classical electrophysiological neuromarkers of motor imagery during real-time cursor control, namely the suppression of sensorimotor mu/beta band-power (8–30 Hz) in contralateral versus ipsilateral imagery [34]. To do so, we first computed the mu- and beta-band power spectral density for all participants (Fast Fourier Transform; Matlab, Mathworks, Natick, Massachusetts, USA) during the cursor control period separately for left and right imagery trials and in electrodes lying in close proximity to sensorimotor hand regions (C3 and C4) as well as in a central control electrode (FCz). Next, we computed the log power ratio (LPR) of contralateral versus ipsilateral imagery trials and took the mean across participants (Fig 3B). Thus, the LPR indicates the relative power modulation between right imagery trials as compared to left imagery trials (at electrode C3) and left imagery trials as compared to right imagery trials (at electrode C4). The LPR at the control electrode was taken as the ratio of left imagery versus right imagery. Log power ratios (LPR) are approximately log-normal, and since a LPR of 0 indicates no difference in the power spectral density (PSD) between contralateral and ipsilateral motor imagery trials, we performed two-tailed t-tests (Bonferonni corrected for multiple comparisons) of the LPR distributions against a distribution with mean 0.

## Results

### Sense of agency (study 1)

**Fig 4:**
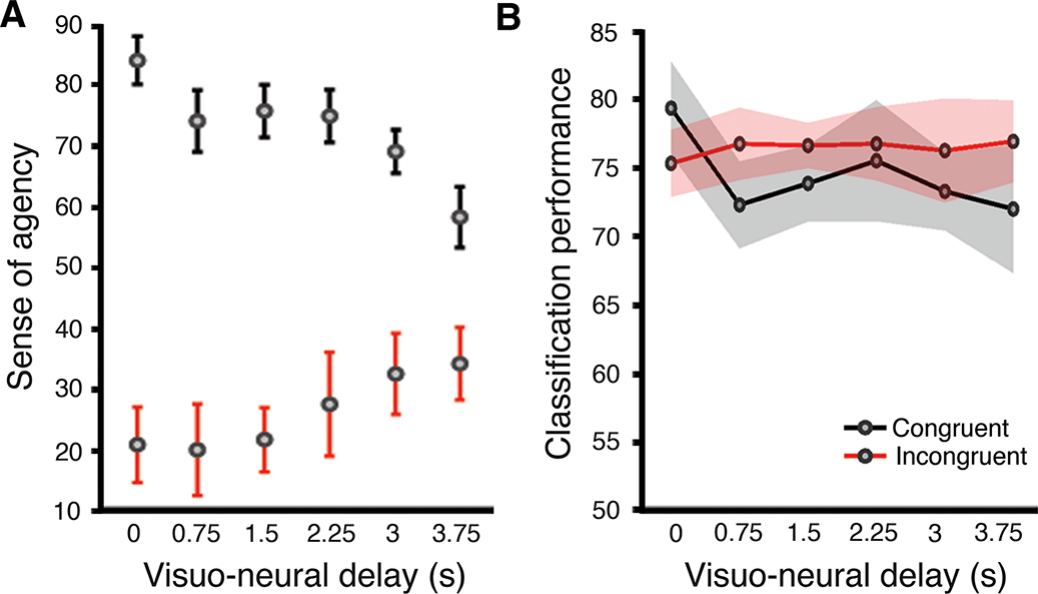
Brain-machine actions are associated with a sense of agency (SOA) that is modulated by neuro-visual delay. (A) SOA (% yes answers) for congruent (black) and incongruent (red) BMI-actions plotted as a function of neuro-visual delay. (B) Classification performance (% real-time decoder performance) remained constant across experimental conditions. Error bars and shaded regions indicate SE of the mean.

The SOA was higher for congruent than for incongruent feedback (main effect of congruence: *F*_(1,7)_ = 43.3; *P* < 0.001; Fig 4A). Furthermore, the SOA depended differently on delay for congruent and incongruent trials as reflected by a delay × congruence interaction (*F*_(5,7)_ = 5.76; *P* = 0.001). For congruent trials, the SOA decreased with increasing delay (from 84% to 58%; *F*_(5,7)_ = 5.33; *P* = 0.001). By contrast, for incongruent trials, the SOA was low and did not depend on neuro-visual delay (20% to 34%; *F*_(5,7)_ = 1.87; *P* = 0.13; see Table 1 for full statistical results). To further analyze the effect of delay on the SOA, we also performed linear regression separately on congruent and incongruent trials and found that SOA decreases with increasing delay for congruent (*r* ^2^ = 0.25, *P* < 0.001), but not for incongruent trials (*r* ^2^ = 0.08, *P* > 0.05). In summary, these results indicate that subjects felt a higher SOA for congruent BMI-actions and that this SOA decreased as a function of neuro-visual delay. On the other hand, directional incongruence led to a large decrease in the SOA and for these trials, the SOA was insensitive to neuro-visual delay.

**Table 1:** Statistical results for sense of agency measures in study 1 and study 2.

Study 1	F / t	P	Study 2	F / t	P
*2 x 6 ANOVA*			*1 x 6 ANOVA*		
Interaction	5.76	0.001	Delay effect	7.817	0.0001
Congruency	43.3	< 0.001	(congruent trials)		
Delay	—	> 0.6			
*1 x 6 ANOVA*			*T-Tests*		
Delay effect			0 > 3.75s		
(congruent trials)	5.33	0.001	0.25 > 3.75s	all	all
			0.5 > 3.75s	> 2.86	< 0.03
			0.75 > 3.75s		
			1.0 > 3.75s		
*1 x 6 ANOVA*					
Delay effect					
(incongruent trials)	1.87	0.125			
*T-Tests*					
congruent > incongruent	all	all			
(at all delays)	> 3.77	< 0.007			
*T-Tests*					
(within congruent)					
0 > 3s	all	all			
0 > 3.75s	> 2.86	< 0.03			
1.5 > 3.75s					
2.25 > 3.75s					
3 > 3.75s					

All statistical contrasts used to assess differences across experimental conditions in SOA judgments (see *Methods*for details). For all pairwise T-test contrasts, only significant differences (*P* < 0.05) are reported.

### Classification performance and EEG analysis (study 1)

We analyzed BMI classification performance across participants and found that it remained significantly higher than chance levels (76.7 ± 8.77 vs. 53.4% ± 1; *mean* ± *SD*; *P* < 0.001; *one-tailed T-test*) in all conditions and exhibited no significant difference across the experimental conditions (*F*_(5,7)_ = 1.36; *P* > 0.25; *two-way ANOVA*; Fig 4B). In order to test for learning effects, we additionally analyzed classification performance across participants as a function of trial order and found no significant effect of trial order (*F*_*(7*,*194)*_ = *0*.*96; P = 0*.*64; one-way ANOVA*), suggesting that the initial steep part of the learning curve had already been traversed during the training sessions.

To investigate whether participants modulated the expected mu-/beta-band oscillations over bilateral sensorimotor cortex to control the cursor movements, we first projected the statistically derived classification features (common spatial patterns) onto the topographical EEG maps for each participant. The resulting spatial filters revealed the importance of electrodes C3 and C4 (located over left and right sensorimotor hand regions) in discriminating between left and right motor imagery, for all individuals (Fig 3A). Next, we performed spectral analysis which showed mu- and beta-band power to be suppressed at both electrode C3 (-1.08 ± 0.24; *mean±SEM*; *two-tailed T-test; P = 0*.*003*) and electrode C4 (-1.02 ± 0.33; *P = 0*.*018*) in contralateral imagery trials as compared to ipsilateral trials, but not at control electrode FCz (0.098 ± 0.14; *P = 0*.*5*; Fig 3B). Taken together, these classification performance and EEG results show that the differences observed in the SOA between our experimental conditions cannot be accounted for by differences in decoder performance, and that the BMI-action was generated by sensorimotor networks classically recruited during motor imagery.

### Cognitive or postdictive mechanisms and the SOA for BMI-actions: SOA as a function of the percentage of time the cursor displaced in the cued direction (study 1)

**Fig 5:**
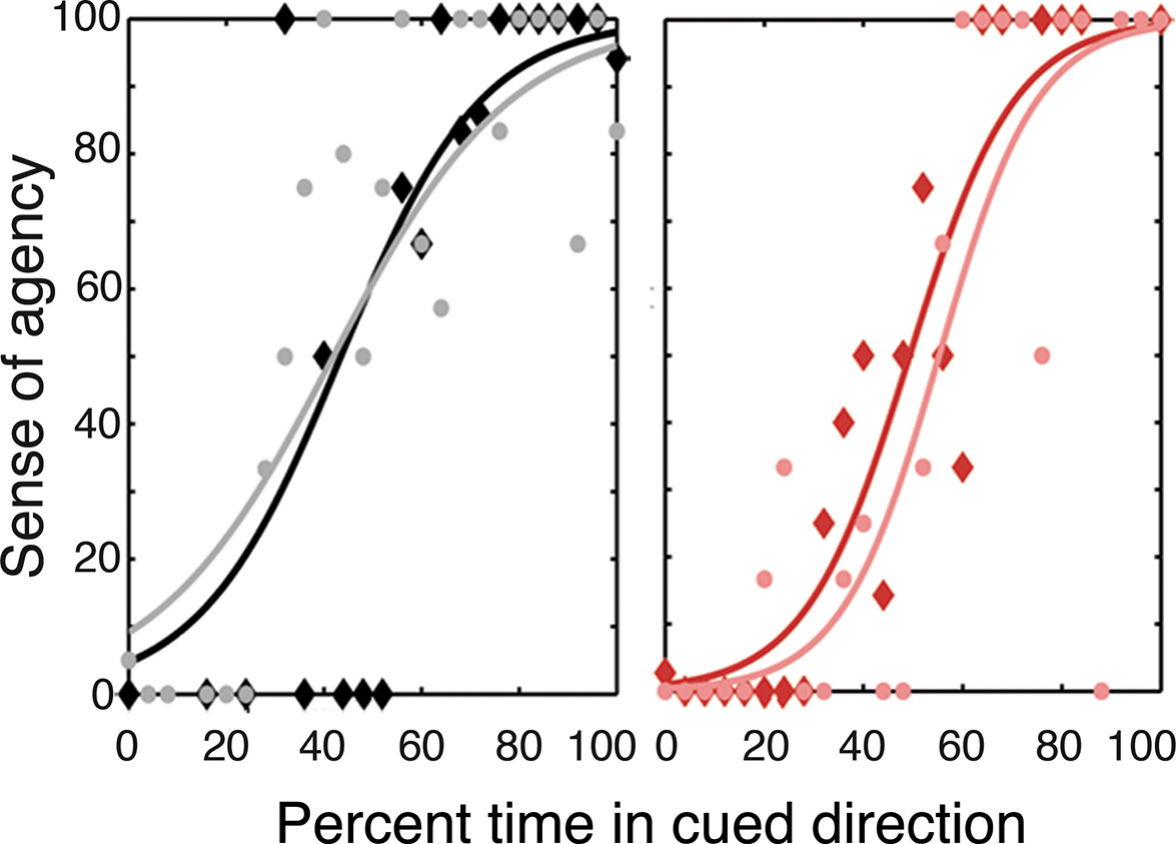
Sense of agency (SOA) for BMI-actions depends on the match between one’s intended direction and the resultant sensory consequences. Psychometric SOA curves (best-fit logistic regression) are plotted as a function of the percentage of time the cursor spent moving in the cued direction for zero-delay congruent (black), high-delay congruent (grey), zero-delay incongruent (red), high-delay incongruent (pink) trials. Data points represent mean SOA across all trials for all participants in 4% bins (of percent time in cued direction).

To explore whether participants’ SOA was influenced by the visual feedback irrespective of BMI performance, we performed a trial-by-trial psychometric analysis between the SOA and the percentage of time that the cursor was displaced in the cued direction. We generated psychometric SOA curves for the pooled, group data and fit them to the time percentage for the two extreme delay conditions (see *Materials and Methods: Trial by Trial Psychometric Analysis*). This analysis revealed that participants have a high SOA when the visual cursor frequently moves in the cued direction and a low SOA when the cursor rarely moves in the cued direction (Fig 5). This relationship held for all tested BMI-actions, that is, for congruent non-delayed (PSE: 62.2; slope: 0.019), congruent delayed (PSE: 62; slope: 0.014), incongruent non-delayed (PSE: 68.2; slope: 0.015), and incongruent delayed (PSE: 66.7; slope: 0.018) trials. No difference in slope or PSE was found across the experimental conditions (all one-tailed bootstrap tests; *P* > 0.05).

### SOA for BMI-actions: Classification performance or visual feedback? (study 1)

Finally, we assessed whether BMI classification performance was a contributing factor to the SOA, or if the SOA was rather based on the congruence of the visual feedback with the cued direction. To this end, we performed a stepwise regression (fixed-effects model) to see if any residual variance in the SOA curves could be accounted for by classification performance. This analysis revealed that classification performance accounted for only 0.07% of the residual variance in SOA judgments, confirming that the visual feedback dominates the SOA for BMI-actions.

### SOA for “BMI-actions” where the sensory consequences are decoupled from brain activity

**Fig 6:**
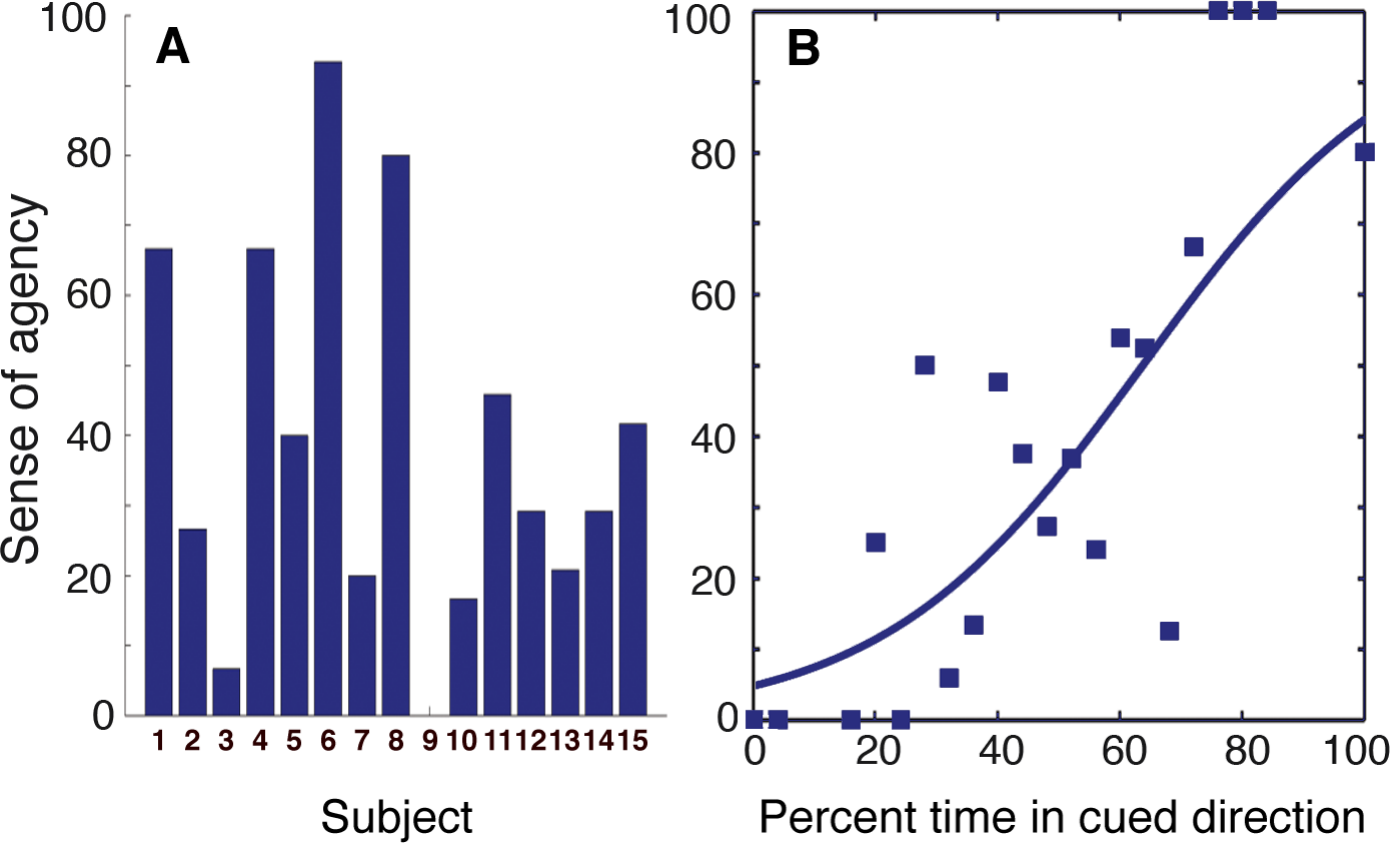
Sense of agency (SOA) for brain-machine actions where sensory consequences are generated independently to brain activity (random-feedback condition). (A) SOA in the random-feedback condition for all subjects across studies 1 and 2 (study 1: subjects 1–8; study 2: subjects 9–15). Individuals reported a wide range of feeling in control over the cursor that moved independently to their brain activity. (B) Psychometric fit (point of subjective equality: 65%) for SOA as a function of the percentage of time the cursor moved in the cued direction. Data points represent mean SOA across all random-feedback condition trials for all participants (in studies 1 and 2) in 4% classification performance or percentage in cued direction bins. Considered alongside Fig 5, these results suggest that SOA for BMI-actions relies on the match between the sensory outcomes (visual cursor position) to one’s intended (cued) direction, rather than to one’s ability to reliably generate the learned motor activity patterns (classification performance).

Analysis of the ‘random-feedback’ condition, where the cursor was displaced independently with respect to the decoded motor signals, revealed that the SOA on these trials was highly variable across individuals (38.89 ± 27.15; *mean ± SD*; Fig 6A). Classification performance for this condition, however, remained high and did not differ from the main experimental conditions in studies 1 and 2 (79.92 ± 9.87; *two-tail T-test*; all *P > 0*.*05*), indicating that the observed changes in the SOA could not have been due to differences in decoder performance.

For these random-feedback trials, the psychometric fit for the percentage of time in the cued (intended) direction versus the SOA again revealed a clear relationship: when the cursor rarely moved in the cued direction, the SOA was low, and when it moved often in the cued direction, the SOA was high (PSE: 65%; Fig 6B).

Taken together, these psychometric analyses demonstrate that the SOA is highly variable for BMI-actions where sensory feedback is decoupled from the decoded motor signals and that this SOA is best predicted by the coherence of the visual feedback with the intended (cued) direction, rather than by a participant’s trial-by-trial classification performance. This again highlights the predominance of the visual feedback in accounting for the SOA for the BMI-actions used in this context.

### Sense of agency (study 2)

**Fig 7:**
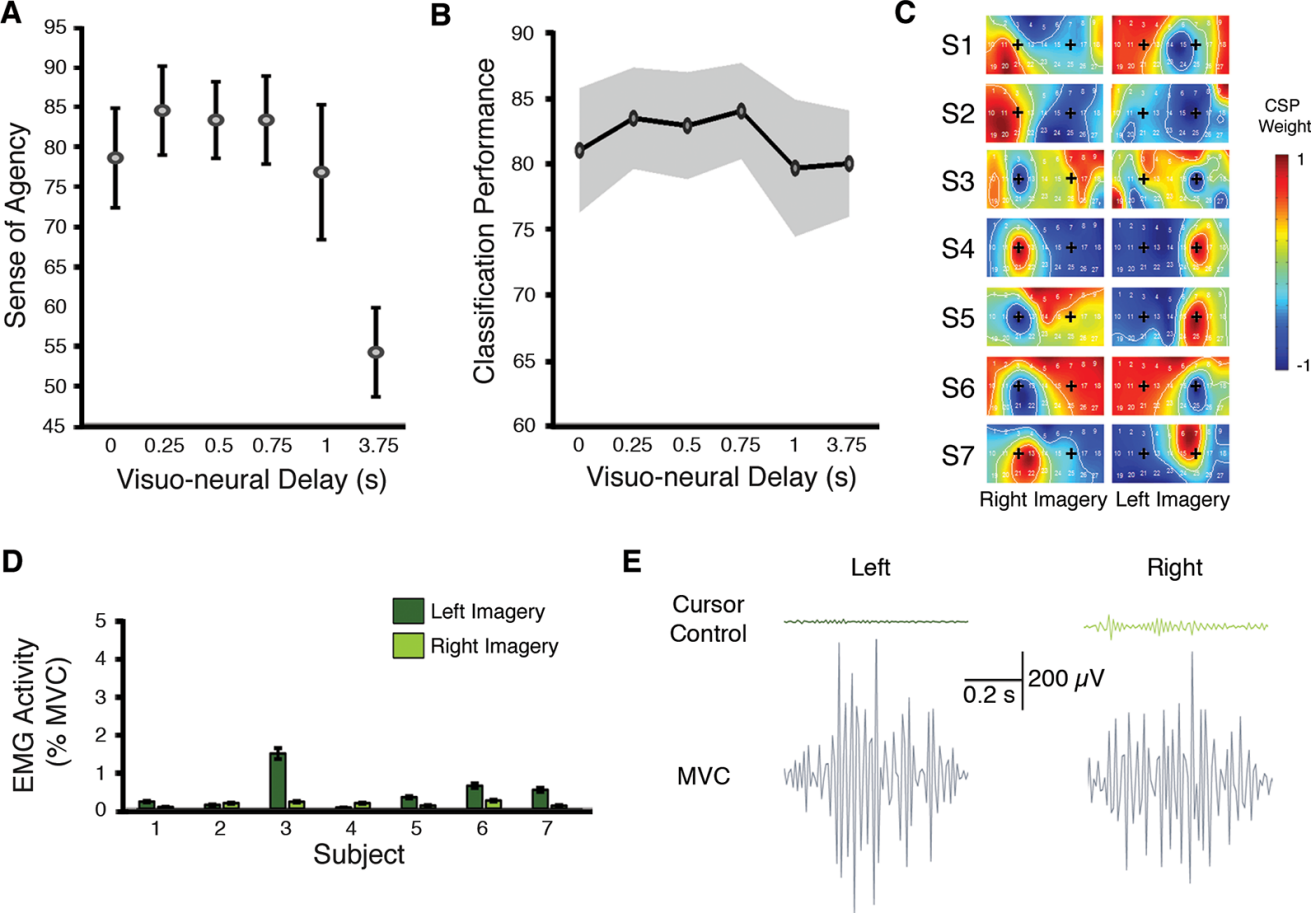
Study 2 results. (A) Sense of agency and (B) classification performance as a function of neuro-visual delay. (C) Per-subject, per-channel, normalized spatial filter weights for each electrode during left and right motor imagery for study 2. On each topographic map, ‘+’ labels are placed over electrodes C3 and C4 for orientation. (D) Mean electromyographic (EMG) activity of left and right forearm flexor muscles during motor imagery. Activity was averaged across trials and conditions for each subject and a ratio was computed to their maximum voluntary contraction (MVC), resulting in very low ratios (all < 2%). (E) Sample EMG traces from participant 2 during left and right cursor control and during MVC trials. In all panels, error bars and shaded regions indicate SE of the mean.

Analysis on the shorter neuro-visual delays tested in study 2 confirmed the effect of delay on the SOA (main effect of delay: *F*_(5,6)_ = 7.82; *P* = 0.0001; Fig 7A). However, neuro-visual delays below 1s did not modulate the SOA for the BMI-actions we tested (all pairwise tests *P* > 0.05).

### Classification performance, EEG, and EMG analysis (study 2)

In study 2, classification performance did not vary across the tested neuro-visual delays (81.70 ± 10.78; *mean ± SD*; *F*_*(1*,*6)*_ = *0*.*19; P* = *0*.*97; one-way ANOVA*; Fig 7B). Furthermore, no difference was found in classification performance as a function of trial order (*F*_(6,167)_ = *0*.*88; P > 0*.*84; one-way ANOVA*).

As for study 1, we confirmed that BMI control was driven by mu-/beta-band modulation over bilateral sensorimotor cortex by projecting the spatial filter weights as scalp topographies. For all individuals, electrodes C3 and C4, located over left and right hand motor areas, respectively, showed the greatest influence of discrimination between left and right hand motor imagery (Fig 7C).

Finally, analysis of the EMG data revealed low muscle contraction ratios for all participants (Fig 7D) that were not significantly different from periods of rest (P < 0.01; *two-tailed T-test*), suggesting that the SOA judgments for BMI-actions were not associated with small limb movements or covert muscle contractions. For illustrative purposes, Fig 7E displays single EMG traces during cursor control (motor imagery) and voluntary overt movements (MVC) for participant 2 in characteristic left- and right-cued imagery trials.

## Discussion

Based on work from the cognitive neuroscience of action awareness and motor control, we introduced spatio-temporal conflicts between decoded cortical motor signals and their resultant sensory consequences during the real-time brain-control of a visual cursor (here defined as BMI-actions). In two experiments, we found that congruent BMI-actions were associated with a robust SOA that was perturbed by the insertion of neuro-visual delay. Classification performance was high across the experimental conditions and the cursor was controlled by mu- and beta-band oscillations (8–30 Hz) over bilateral sensorimotor cortex in the absence of overt muscle activity.

Although it is well described that bodily actions are associated with a SOA that is reduced by motor-visual [14], motor-auditory [12], and motor-somatosensory [35] delays between the bodily action and its sensory outcomes, this is the first study (to our knowledge) to target the SOA for BMI-actions. Investigating the SOA in this novel, ‘disembodied’ context avoids some of the confounds of earlier work. In particular, bodily actions are normally accompanied by re-afferent proprioceptive and tactile signals that were absent in the present experiments, as BMI-actions are generated without involvement of the musculoskeletal system [41].

Despite this unique context, our analyses showed that congruent BMI-actions are associated with a robust SOA that decreases with increasing discrepancy between the predicted and actual sensory feedback, just as for bodily actions. This suggests that the SOA for these two types of actions may be based on common brain mechanisms and may recruit some of the same regions that have been described for the SOA for bodily actions, including the supplementary motor area, ventral premotor cortex, posterior parietal cortex, temporo-parietal junction, and cerebellum (reviewed in [2]).

We also observed differences between the SOA for congruent BMI- and bodily actions, suggesting distinct brain mechanisms for these types of actions. For instance, the present data show the SOA for congruent BMI-actions without neuro-visual delay to be ~85%. Most studies report higher SOA values for bodily actions in comparable experimental conditions [4,12,14]. Moreover, the temporal sensitivity of the SOA for congruent BMI-actions was lower than for bodily actions: study 2 revealed that participants were insensitive to neuro-visual delays of less than 1s, whereas previous SOA studies for bodily actions have reported that motor-visual or audio-motor delays of 150–300 ms can significantly perturb the SOA [12,14,32]. The most likely explanation for these differences is the longer latency inherent in non-invasive BMIs between the control signals in the brain (modulation of mu and beta rhythms) and the end effector. Moreover, the signal-to-noise ratio (*i*.*e*. overall end-effector control reliability) is much lower for BMI-actions than for the overlearned bodily actions of previous SOA studies. The low signal-to-noise ratio for BMI-actions might arise due to the lack of tactile-proprioceptive feedback, imperfections implicit to the selected neural decoding process, subject selection (although we tried to include only those subjects who were able to control the BMI), and the novelty of a motor imagery task. Indeed, recent work demonstrated that the SOA for bodily actions increases with learning of novel overt motor tasks [42].

By assessing classification performance (Fig 3B) alongside the SOA (Fig 3A), we observed that participants generated similar mu-/beta-band oscillations in sensorimotor cortex in all experimental conditions, even though the SOA changed as a function of congruence and neuro-visual delay. This dissociation between the SOA and classification performance reinforces our conclusion that the SOA was not based on a comparison between the mu-/beta-band oscillations in sensorimotor cortex and brain signals associated with the visual feedback. Considering this, how could our participants form a SOA? Postdictive SOA theories hold that the SOA is based on comparing cognitive cues (*e*.*g*. intentions and goals) with the sensory outcomes [22]. In the present context, a high SOA for poorly controlled BMI-actions could occur if the cursor displacements were to match the subject’s cued (and likely intended) direction.

Our paradigm, and in particular the incongruent and ‘random-feedback’ BMI-actions, allowed us to test this special situation where a mismatch between the decoded brain signals and the sensory consequences yields congruence between the cued cursor direction and the actual cursor displacement. Indeed, our trial-by-trial analyses revealed, for all experimental conditions, a distinct relationship between the SOA and the match of the visual feedback with the cued direction (Fig 5). This result is compatible with the proposal that the SOA depends on cognitive agency signals and may be independent of the motor imagery-based signals in sensorimotor cortex. This interpretation is further supported by our SOA analysis in the ‘random-feedback’ condition (Fig 7), where participants could form a high SOA for cursor movements if the cursor tended to move toward the cued direction. Collectively, these findings suggest that the SOA for BMI-actions is largely reliant on the comparison of the re-afferent visual cues with visual feedback rather than with the motor signals used to control the BMI.

Our regression analyses did not yield clear evidence that internal predictive models [18–20] played a role in the SOA judgments since the vast majority of the variance in SOA judgments could be accounted for by the visual feedback alone. It might be that sensory feedback dominates the SOA in the context of novel or unreliable cognitive operations such as motor imagery in non-invasive BMIs. Thus, such predictive signals may be weak and easily overridden due to the dominance of the visual feedback. Further research is required to assess the relative roles of sensory feedback versus predictive models of sensorimotor brain activity in BMI-actions. In future experiments, researchers may test populations of skilled BMI users, perform more training with the participants, or investigate the SOA for BMI-actions performed in the absence of visual feedback.

In conclusion, we argue that cognitive, postdictive agency theories rather than predictive best account for congruent, incongruent, and ‘random-feedback’ EEG-based BMI-actions. Alternatively, recent multifactorial theories [3, 32, 43–47], allow for relative contextual weighting of predictive and postdictive information in the formation of the SOA. This unified framework has been used to explain how the SOA can emerge for joint actions in human dyads [42], a situation that may be analogous to the “joint” action between human and machine needed to generate BMI-actions. Our findings, which show that some combination of visual feedback and internal monitoring of sensorimotor activity best explains the SOA for BMI-actions, fit favorably within such a framework. Further research will be required in order to better understand the relative weighting of predictive and postdictive cues in forming the SOA for BMI-actions and differences in weighting with respect to the SOA for bodily actions. For instance, it could be the case that for bodily actions, predictive mechanisms play a much larger role in determining the SOA, whereas for BMI-actions, postdictive mechanisms dominate in the presence of sensory feedback.

In our quest for repair, substitution, and augmentation, we will likely witness an increase in the use of brain-controlled technologies [29,30]. Experimental setups such as the present one allow for the investigation of the SOA for these new types of “actions” and provide empirical, neuroscience-based data for the unique ethical and moral challenges raised with respect to responsibility for machine-controlled actions [48–50].
